# Functional Characterization of Two Putative DAHP Synthases of AroG1 and AroG2 and Their Links With Type III Secretion System in *Ralstonia solanacearum*

**DOI:** 10.3389/fmicb.2019.00183

**Published:** 2019-02-12

**Authors:** Weiqi Zhang, Jing Li, Xiaojun Shi, Yasufumi Hikichi, Yong Zhang, Kouhei Ohnishi

**Affiliations:** ^1^College of Resources and Environment, Southwest University, Chongqing, China; ^2^The Ninth People’s Hospital of Chongqing, Chongqing, China; ^3^Laboratory of Plant Pathology and Biotechnology, Kochi University, Kochi, Japan; ^4^Research Institute of Molecular Genetics, Kochi University, Kochi, Japan

**Keywords:** *Ralstonia solanacearum*, DAHP synthase, AroG, type III secretion system, pathogenesis

## Abstract

Type three secretion system (T3SS) is essential for *Ralstonia solanacearum* to cause disease in host plants and we previously screened AroG1 as a candidate with impact on the T3SS expression. Here, we focused on two putative DAHP synthases of AroG1 and AroG2, which control the first step of the shikimate pathway, a common route for biosynthesis of aromatic amino acids (AAA), to characterize their functional roles and possible links with virulence in *R. solanacearum*. Deletion of *aroG1/2* or *aroG1*, but not *aroG2*, significantly impaired the T3SS expression both *in vitro* and *in planta*, and the impact of AroG1 on T3SS was mediated with a well-characterized PrhA signaling cascade. Virulence of the *aroG1/2* or *aroG1* mutants was completely diminished or significantly impaired in tomato and tobacco plants, but not the *aroG2* mutants. The *aroG1/2* mutants failed to grow in limited medium, but grew slowly *in planta*. This significantly impaired growth was also observed in the *aroG1* mutants both *in planta* and limited medium, but not in *aroG2* mutants. Complementary *aroG1* significantly restored the impaired or diminished bacterial growth, T3SS expression and virulence. Supplementary AAA or shikimic acid, an important intermediate of the shikimate pathway, significantly restored diminished growth in limited medium. The promoter activity assay showed that expression of *aroG1* and *aroG2* was greatly increased to 10-20-folder higher levels with deletion of the other. All these results demonstrated that both AroG1 and AroG2 are involved in the shikimate pathway and cooperatively essential for AAA biosynthesis in *R. solanacearum*. The AroG1 plays a major role on bacterial growth, T3SS expression and pathogenicity, while the AroG2 is capable to partially carry out the function of AroG1 in the absence of AroG1.

## Introduction

Like in many pathogenic bacteria of animals and plants, the syringe-like type three secretion system (T3SS) is essential for *Ralstonia solanacearum* to cause disease in host plants, which is a causal agent of bacterial wilt disease on more than 450 plant species worldwide (Genin et al., 2005; Genin and Denny, 2012; Jiang et al., 2017). Bacteria use this syringe-like apparatus to inject virulence factors, called type III effectors (T3Es), into host cytosol to subvert host defense and cause diseases (Cunnac et al., 2004; Tasset et al., 2010; Fujiwara et al., 2016; Popa et al., 2016). *R. solanacearum* is heterogeneous and currently regarded as a *Ralstonia solanacearum* species complex (RSSC), while the T3SS is highly conserved in RSSC strains, which is encoded by approximately 20 genes of hypersensitive response and pathogenicity (*hrp*) gene cluster and is globally controlled by a complex regulation network (Cunnac et al., 2004; Valls et al., 2006; Hikichi et al., 2007; Genin and Denny, 2012).

In general, the T3SS and entire T3Es (more than 100 T3Es in RSSC) are directly controlled by a master regulator HrpB, which is an AraC type of transcriptional regulator and binds directly to plant-inducible promoter (PIP) motif in the promoter regions of its target genes (Cunnac et al., 2004; Mukaihara et al., 2004, 2010). Two close paralogs of OmpR/PhoB family of two-component response regulators, HrpG and PrhG, positively regulate the *hrpB* expression in a parallel manner (Plener et al., 2010; Zhang et al., 2013). Expression of the *hrpB* and T3SS is not activated until the bacterium gets contact with host signals or some mimic signals, such as in nutrient- limited media that mimic plant apoplastic fluids (Arlat et al., 1992; Angot et al., 2006; Yoshimochi et al., 2009b). These signals are presumed to be recognized by an outer membrane protein PrhA and transferred to HrpG by a well-characterized signaling cascade of PrhA-PrhR/I-PrhJ or some novel signaling cascades (Marenda et al., 1998; Genin et al., 2005; Yoshimochi et al., 2009a; Zuluaga et al., 2013). Host signals can greatly increase the T3SS expression to about 10–20-folder higher levels than that in nutrient-limited media (Marenda et al., 1998; Yoshimochi et al., 2009b). HrpG and PrhG can respond to host signals by phosphorylation at certain residues and in turn activate the *hrpB* expression, while their regulation mechanism remains to be further elucidated (Yoshimochi et al., 2009b; Zhang et al., 2013). Moreover, a LysR type of transcriptional regulator PhcA negatively regulates *hrpG* expression, which is activated at high cell density and binds to promoters of *prhI/R* genes to repress their expression, and in tandem to repress the *hrpB* expression, while the PhcA positively regulates *prhG* expression (Genin et al., 2005; Yoshimochi et al., 2009a; Plener et al., 2010; Zhang et al., 2013). This results in dual regulation pathways of PhcA on *hrpB* expression, and *R. solanacearum* might switch from using the HrpG to PrhG for *hrpB* activation in a cell density-dependent manner (Zhang et al., 2013).

In order to further elucidate the global regulation on T3SS in *R. solanacearum*, we generated a *popA-lacZYA* fusion to monitor expression profiles of the T3SS in OE1-1, and screened several candidates with impact on expression of the T3SS by transposon mutagenesis, including the AroG1 (Rsc2660 in GMI1000) (Zhang et al., 2013), which is annotated as a putative DAHP synthase and catalyzes the formation of 3-deoxy-D- arabino-heptulosonate-7-phosphate (DAHP) by condensation of phosphoenolpyruvate (PEP) and erythrose 4-phosphate (E4P) (Ogino et al., 1982; Herrmann, 1983). This is the first step in the shikimate pathway that comprises seven steps beginning with the condensation of PEP and E4P, and ending with the formation of chorismate, and is a common route for biosynthesis of aromatic amino acids (AAA), including L-phenylalanine (Phe), L-tyrosine (Tyr) and L-Tryptophan (Trp), in bacteria, fungi, plants, and some protists (Herrmann and Weaver, 1999; Sprenger, 2006; Maeda and Dudareva, 2012). Chorismate is a common precursor for individual postchorismate pathways that lead to biosynthesis of AAA and their derivatives, such as vitamin K, ubiquinone and folic acid (Bentley, 1990; Dosselaere and Vanderleyden, 2001; Gosset et al., 2001). To date, all the enzymes, their corresponding genes and metabolic intermediates in the shikimate pathway have been well characterized in Gram-negative *Escherichia coli* and Gram-positive *Bacillus subtilis* (Pittard and Yang, 2005; Sprenger, 2006). A total of three isoenzymes of DAHP synthases of AroF, AroG and AroH, have been identified in *E. coli*, expression of which is inhibited by their corresponding production of AAA, while only one DAHP synthase is identified in *B. subtilis*, expression of which is not affected by its corresponding production of AAA (Umbarger, 1978; Herrmann, 1983; Pittard, 1996; Wu et al., 2005).

With genome searching^1^, only AroG, including two putative DAHP synthases of AroG1 and AroG2 (Rsc0743 in GMI1000) is annotated in RSSC, which share 58% of identities at amino acids (AA), and are greatly conserved in RSSC. As a vascular phytopathogenic bacterium, extensive proliferation in xylem vessels and its producing extracellular polysaccharide (EPS) slime have been believed to be the other main virulence factors in *R. solanacearum*, which severely block the sap flow in xylem vessels and causes plants rapidly stunting and wilting (Roberts et al., 1988; Denny, 1995; Vasse et al., 1995). In addition to T3SS and EPS, several molecular determinants are also involved in pathogenicity of *R. solanacearum* (Genin and Denny, 2012). Here, we focused on these two putative DAHP synthases of AroG1 and AroG2 to characterize their functional roles in AAA biosynthesis and possible links with virulence in *R. solanacearum*.

## Materials and Methods

### Bacterial Strains and Culture Conditions

*Ralstonia solanacearum* strains used in this study are listed in Table 1, which are derivatives of two typical strains of OE1-1 and GMI1000. OE1-1 is virulent on both tomato and tobacco plants (Kanda et al., 2003), while GMI1000 is virulent on tomato plants but elicits HR in tobacco leaves (Salanoubat et al., 2002). *R. solanacearum* strains were grown at 28°C in nutrient-rich medium (B medium) or nutrient-limited medium (sucrose medium, *hrp-*inducing medium) (Yoshimochi et al., 2009b). *E. coli* DH12S and S17-1 were grown in Luria-Bertani medium at 37°C for plasmid construction and conjugational transfer, respectively.

**Table 1:** Bacterial strains used in this study.

Strain	Relative characteristics	References
OE1-1	Wild-type, race 1, biovar 3	Kanda et al., 2003
RK5043	OE1-1, *phcA-lacZYA*	Yoshimochi et al., 2009b
RK5046	OE1-1, *hrpB-lacZYA*	Yoshimochi et al., 2009b
RK5050	OE1-1, *popA-lacZYA*	Yoshimochi et al., 2009b
RK5120	OE1-1, *hrpG-lacZYA*	Yoshimochi et al., 2009b
RK5124	OE1-1, *prhJ-lacZYA*	Yoshimochi et al., 2009b
RK5130	OE1-1, *prhIR-lacZYA*	Yoshimochi et al., 2009b
RK5134	OE1-1, *prhA-lacZYA*	Yoshimochi et al., 2009b
RK5212	OE1-1, *prhG-lacZYA*	Zhang et al., 2013
RK5619	OE1-1, *prhN-lacZYA*	Zhang et al., 2015
RQ5687	*popA-lacZYA, ΔaroG1*	This study
RQ5803	*popA-lacZYA*, Δ*aroG2*	This study
RQ5806	*popA-lacZYA*, Δ*aroG1/2*	This study
RQ5838	*hrpB-lacZYA*, Δ*aroG1*	This study
RQ5840	*hrpG-lacZYA*, Δ*aroG1*	This study
RQ5842	*prhG-lacZYA*, Δ*aroG1*	This study
RQ5930	OE1-1, Δ*aroG1*	This study
RQ5963	OE1-1, Δ*aroG2*	This study
RQ6011	OE1-1, Δ*aroG1/2*	This study
RQ6150	*prhIR-lacZYA*, Δ*aroG1*	This study
RQ6153	*prhJ-lacZYA*, Δ*aroG1*	This study
RQ6156	*prhA-lacZYA*, Δ*aroG1*	This study
RQ6159	*prhN-lacZYA*, Δ*aroG1*	This study
RQC226	RK5050, Δ*aroG1* + *aroG1*	This study
RQC227	RK5050, Δ*aroG1/2* + *aroG1*	This study
RQC212	OE1-1, *aroG1-lacZYA*	This study
RQC214	OE1-1, *aroG2-lacZYA*	This study
RQC216	OE1-1, Δ*aroG1, aroG1-lacZYA*	This study
RQC218	OE1-1, Δ*aroG2, aroG1-lacZYA*	This study
RQC228	OE1-1, Δ*aroG1, aroG2-lacZYA*	This study
RQC220	OE1-1, Δ*aroG2, aroG2-lacZYA*	This study
RQC264	OE1-1, Δ*aroG1/2, aroG1-lacZYA*	This study
RQC260	OE1-1, Δ*aroG1/2, aroG2-lacZYA*	This study
GMI1000	Wild-type, race 1, biovar 4	Salanoubat et al., 2002
GF0001	GMI1000, *popA-lacZYA*	Zhang et al., 2018
GF0032	GF0001, Δ*aroG1*	This study
GF0033	GF0001, Δ*aroG2*	This study
GF0034	GF0001, Δ*aroG1/2*	This study
RQC213	GF0001, Δ*aroG1* + *aroG1*	This study
RQC215	GF0001, Δ*aroG1/2* +*aroG1*	This study
RQC258	RK5050, Δ*aroG1* + *g1p::G2*	This study
RQC259	RK5050, Δ*aroG1/2* + *g1p::G2*	This study
RQC260	RK5050, Δ*aroG1* + *g2p::G1*	This study
RQC261	RK5050, Δ*aroG1/2* + *g2p::G1*	This study
RQC286	RK5050, Δ*aroG1* + *aroG2-N*	This study


### Mutants Generation With In-Frame Deletion of *aroG1* and *aroG2*

In this study, mutants with in-frame deletion of target genes were generated with the pK18mobsacB based homologue recombination (Zhang et al., 2011). For plasmid construction, two DNA fragments (about 600-bp) flanking the target gene were PCR amplified from OE1-1 genomic DNA with respective primer-pairs, and subjected for joint PCR to generate DNA fragment, in which coding sequence (CDS) of target gene was absent. These DNA fragments were finally sub-cloned into pK18mobsacB to get pk18daroG1 and pk18daroG2, respectively. After validating sequence, these plasmids were transferred from *E. coli* S17-1 into *R. solanacearum* strains by conjugation and the *aroG1* and *aroG2* mutants were generated with confirmation of colony PCR. The *aroG1* was further deleted from *aroG2* mutants to generate *aroG1/2* mutants. Primers used in this study were listed in Supplementary Table S1.

### Complementation Analyses

In this study, genetic complementation was performed with pUC18-mini-Tn7T-Gm based site specific chromosome integration system (Choi et al., 2005; Zhang et al., 2011). For plasmid construction, DNA fragment, containing the *aroG1* and upstream region of about 600-bp (empirically harboring native promoter) was PCR amplified from OE1-1 genomic DNA, and finally sub-cloned into pUC18-mini-Tn7T-Gm to get pUCaroG1. After validating sequence, the complementary *aroG1* was integrated into chromosome of *R. solanacearum* strains at a single attTn*7* site (25-bp downstream of *glmS*) and confirmed by colony PCR (Zhang et al., 2011).

### Mutants Generation With Promoter-Exchanged *aroG1, aroG2* and Truncated AroG2

Mutants with promoter-exchanged *aroG1, aroG2* or truncated AroG2 were generated with the above site specific chromosome integration system. For promoter exchange, upstream regions of *aroG1* and *aroG2* (about 600-bp to start codon, empirically harboring promoter) were PCR amplified from OE1-1 genomic DNA, and subjected for joint PCR to fuse promoter of *aroG2* and *aroG1* with CDS of *aroG1* and *aroG2*, correspondingly. These DNA fragments were finally sub-cloned into pUC18-mini-Tn*7*T-Gm to get pUC*g2p::*G1 (*aroG2* promoter-*aroG1* CDS) and pUC*g1p::*G2 (*aroG1* promoter-*aroG2* CDS), respectively. After validating sequence, these promoter exchanged *aroG1* and *aroG2* were integrated into chromosomes of *aroG1* or *aroG1/2* mutants to generate desired mutants (Table 1).

The unique N-terminal region of 43 AA in AroG2 is one of remarkable difference between AroG1 and AroG2, and we generated mutants with N-terminal truncated AroG2 by integrating truncated *aroG2* into *aroG1* or *aroG1/2* mutants with the above site specific chromosome integration system. Two DNA fragments flanking deletion region (129-bp after start codon) were PCR amplified from OE1-1 genomic DNA, respectively, and subjected for joint PCR to generate desired DNA fragment, in which the region of 129-bp was absent. This DNA fragment was finally sub-cloned into pUC18-mini-Tn*7*T-Gm to get pUCaroG2N. After validating sequence, this truncated *aroG2* was integrated into chromosome of *aroG1* or *aroG1/2* mutants to generate desired mutants (Table 1).

### Generation of Reporter Fusion of *aroG1-lacZYA* and *aroG2-lacZYA* for Promoter Activity Assay

Reporter strains with *aroG1-lacZYA* and *aroG2-lacZYA* were generated with the site specific chromosome integration system. In general, promoter-less *lacZYA* was fused to *aroG1* or *aroG2* at about 54-bp after start codon, in which 6-bp of nucleotide acids were replaced with Kpn I for *lacZYA* insertion. The Kpn I site was generated by PCR primers and the DNA fragment containing promoter region and Kpn I site was firstly cloned into pUC18-mini-Tn7T-Gm and then promoter-less *lacZYA* was inserted to get pUCaroG1-lacZYA and pUCaroG2-lacZYA, respectively. After validating sequence, these reporter fusions were integrated into chromosome of *aroG1* or *aroG1/2* mutants to generate desired mutants (Table 1).

### *β*-Galactosidase Assay

In this study, expression of genes, which were fused with promoter-less *lacZYA*, were evaluated with the *β*-galactosidase assay both *in vitro* and *in planta* as previously described (Zhang et al., 2013). Enzyme activity *in vitro* was expressed in Miller Units (Miller, 1992), and that *in planta* was normalized with luminescence divided by cells number. Each assay was independently repeated for at least four times, and each trial included three replications. Mean values of all experiments were averaged with SD and statistical significance was assessed using a *post hoc* Dunnett test following ANOVA.

### Virulence Assay and HR Test

In this study, tomato plants (*Solanum lycopersicum* cv. Moneymaker) and tobacco plants (*Nicotiana tabacum* CV. Bright Yellow) were grown at 25°C for 2–3 or 3–4 weeks, respectively, and subjected for virulence assay. Tomato plants were inoculated by methods of soil-soaking, which mimics natural invasion through roots, and petiole inoculation, which enables direct invasion into xylems vessels (Yao and Allen, 2007; Zhang et al., 2013). Tobacco plants were inoculated by methods of soil-soaking and leaf-infiltration, which enables direct invasion into host plants (Zhang et al., 2013). For soil-soaking, unwounded plants were inoculated by pouring bacterial suspension onto the soil to a final concentration of 1 × 10^7^ cfu g^-1^ soil. For petiole inoculation, 2 μl of bacteria suspension at 10^8^ cfu ml^-1^ was dropped onto the freshly cut surface of tomato petioles. For leaf-infiltration, about 50 μl of bacterial suspension at 10^8^ cfu ml^-1^ was infiltrated into tobacco leaves. Each assay was repeated independently for at least four times and each trial included 12 plants. Wilt symptoms of plants were inspected as 1–4 disease index and mean values of all experiments were averaged with SD. Disease index was recorded with integer representation and the SD was extremely high. In consideration of aesthetic appearance, the SD was not presented in figures for virulence assay. Statistical significance was assessed using a *post hoc* Dunnett test following ANOVA.

HR test was performed in tobacco leaves. Briefly, approximately 50 μl of bacterial suspension at 10^8^ cfu ml^-1^ was infiltrated into tobacco leaves with a blunt-end syringe and development of necrotic lesions in leaves was recorded periodically (Zhang et al., 2015). Each test was repeated independently at least for four times and each trial included four plants. The representative result was presented.

### Bacterial Growth *in vitro* and *in planta*

Bacterial growth in media, including rich and limited medium was measured with the optical density at 600 nm (OD600). For growth complementation, AAA or shikimic acid (SA) was supplemented into limited medium at a concentration of 0.1 mM and the OD600 was assayed. Growth in tomato stems and tobacco leaves was assessed as described previously (Zhang et al., 2013). Briefly, stem tissues from tomato plants and leaf disks from tobacco plants were removed daily for quantification of cells number by dilution plating. Cells density in stems and leaves was expressed in log_10_ cfu g^-1^ and log_10_ cfu cm^-2^, respectively. Each assay was repeated independently for at least four times and each trial included four plants. Mean values of all experiments were averaged with SD and statistical significance was assessed using a *post hoc* Dunnett test following ANOVA.

## Results

### AroG1 and AroG2 Are Cooperatively Essential for Growth of *R. solanacearum* in Medium, While the AroG1 Plays a Major Role

**FIGURE 1:**
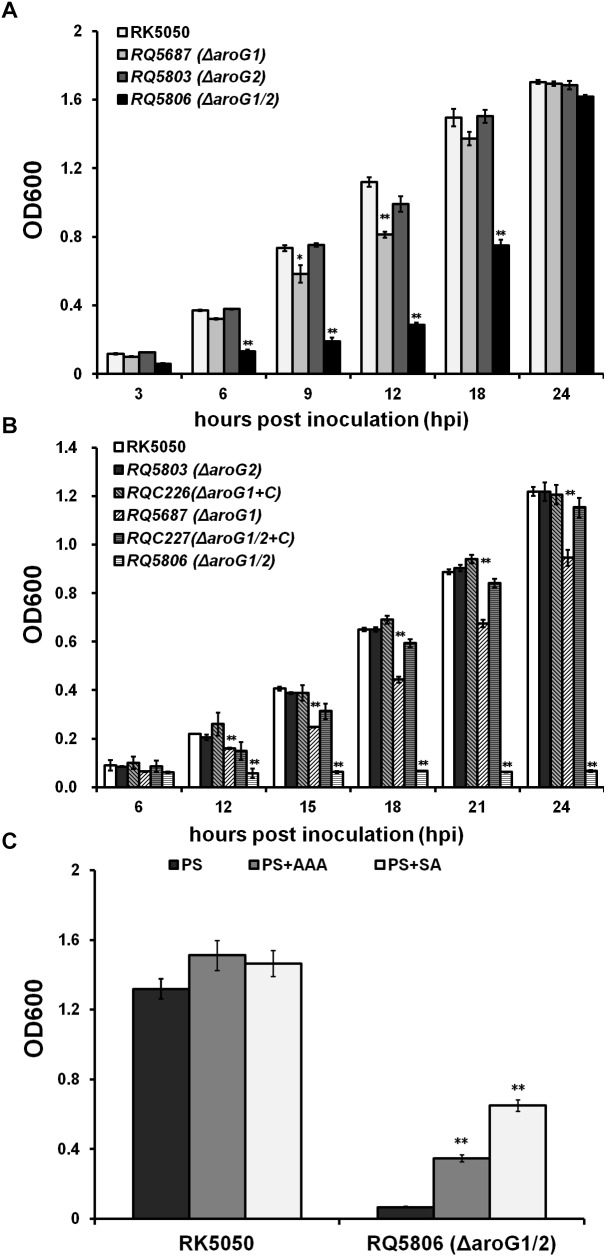
Growth of *aroG* mutants of OE1-1 in media of **(A)** nutrient-rich medium (B medium), **(B)** nutrient limited medium (sucrose medium, PS), **(C)** PS supplemented with AAA or SA. Each of three AAA (L-Phe, L-Tyr, and L-Trp) or SA was added into sucrose medium at a concentration of 0.1 mM. Cells suspension (OD600 = 1.0, washed twice with distilled water) was inoculated into media as a ratio of 1/100, and OD600 was measured periodically. RQC226 and RQC227 refer to *aroG1* and *aroG1/2* mutants complemented with *aroG1* of OE1-1, respectively. Mean values of four independent trials with three replications per trial were averaged and presented with SD (error bars). Statistical significance between RK5050 (wild type) and *aroG* mutants was assessed using a *post hoc* Dunnett test following ANOVA. Significance level, ^∗∗^ indicates *P* < 0.01 (*t*-test).

AroG catalyzes the first step in the shikimate pathway that controls AAA biosynthesis. We therefore evaluated the growth of *aroG* mutants in nutrient rich and limited media. The *aroG2* mutant (RQ5803) exhibited identical growth as RK5050 in both media, while growth of *aroG1* mutant (RQ5687) was significantly impaired in both media (Figure 1A,B). Growth of *aroG1/2* mutant (RQ5806) was much less than that of RQ5687 in rich medium (Figure 1A), while it failed to grow in limited medium, which remained the OD600 at about 0.1 till 24 h post inoculation (hpi) (Figure 1B). Note that growth of *aroG1* mutant and *aroG1/2* mutant could eventually reach to the maximum OD600 as RK5050 at 24 hpi in rich medium (Figure 1A). Complementary *aroG1* fully restored the impaired growth of RQ5687 and diminished growth of RQ5806 to that of RK5050 in limited medium (Figure 1B), indicating that both AroG1 and AroG2 are cooperatively essential for bacterial growth of *R. solanacearum*. The AroG1 plays a major role on growth, while the AroG2 becomes to partially function in the absence of AroG1.

A total of three isoenzymes of DAHP synthase, including AroF, AroG, and AroH, have been identified in some bacteria to date, while only the AroG, including AroG1 and AroG2 are annotated in genomes of RSSC, which are highly conserved in RSSC and share more than 90% identities at AAs, respectively. GMI1000 is well known to be different from OE1-1 on tobacco plants, and we ascertained whether AroG1 and AroG2 played roles on growth of GMI1000. Consistent with that of OE1-1, the *aroG2* mutant (GF0033) exhibited identical growth as GMI1000 in both media, while growth of *aroG1* mutant (GF0032) was significantly impaired both in rich medium (data not shown) and sucrose medium (Supplementary Figure S1). Growth of *aroG1/2* mutant (GF0034) was also significantly impaired in rich medium (data not shown) but diminished in limited medium (Supplementary Figure S1). As expected, the AroG1 of OE1-1 significantly restored diminished growth of GF0034 and fully restored the impaired growth of GF0032 to that of wild- type strain in sucrose medium (Supplementary Figure S1), confirming that the involvement of AroG1 and AroG2 on bacterial growth is conserved in RSSC, and the AroG1 of OE1-1 is functionally equivalent to that of GMI1000.

### Both AroG1 and AroG2 Are Important for AAA Biosynthesis in *R. solanacearum*

AroG controls the first step for AAA biosynthesis, which are essential for bacterial growth, and the *aroG1/2* mutants were auxotrophic in limited medium. We therefore evaluated whether their diminished growth was due to the deficient of AAA. Three AAA, including L-Phe, L-Tyr, and L-Trp, were supplemented into sucrose medium and OD600 was assessed. Supplementary AAA enables the *aroG1/2* mutant (RQ5806) to grow in sucrose medium, even though it could just partially recover the diminished growth, which reached the maximum OD600 of approximately 0.4 at 24 hpi (Figure 1C). SA is an important intermediate in the shikimate pathway, and we evaluated whether supplementary SA could recover the diminished growth of RQ5806. Supplementary SA could also partially restore the diminished growth of RQ5806 to an OD600 of approximately 0.7 at 24 hpi (Figure 1C). All these suggested that both AroG1 and AroG2 are involved in the shikimate pathway, and hence are responsible for AAA biosynthesis in *R. solanacearum*.

### Both AroG1 and AroG2 Are Cooperatively Important for the T3SS Expression *in vitro*, While the AroG1 Plays a Major Role

**FIGURE 2:**
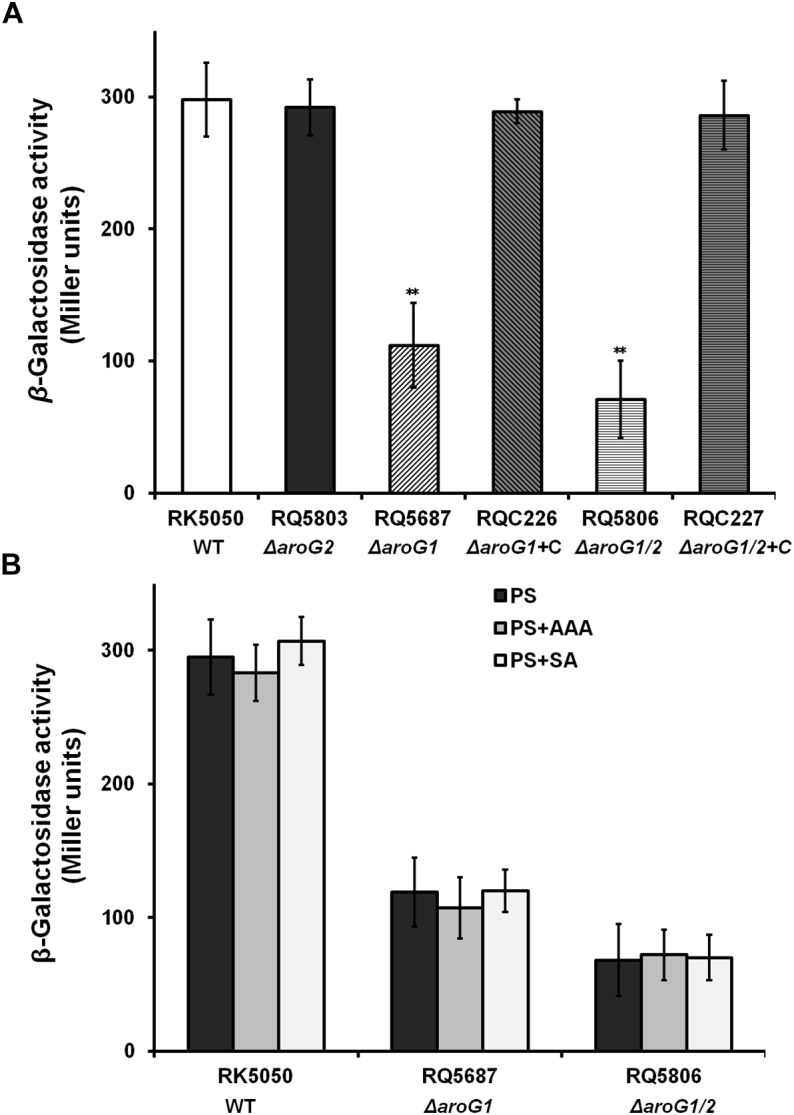
Expression of *popA* in *aroG* mutants of OE1-1 in **(A)** PS and **(B)** PS supplemented with AAA or SA. Cells (OD600 = 1.0) were inoculated into PS medium as a ratio of 1/100, grown to an OD600 of approximately 0.1 and subjected for *β*-galactosidase assay. RQC226 and RQC227 refer to *aroG1* and *aroG1/2* mutants complemented with *aroG1*, respectively. Enzymatic activities were presented in Miller units. Mean values of four independent trials with three replications per trial were averaged and presented with SD (error bars). Statistical significance between RK5050 (wild-type) and *aroG* mutants was assessed using a *post-hoc* Dunnett test following ANOVA. Significance level, ^∗∗^ indicates *P* < 0.01 (*t*-test).

We previously used a *popA-lacZYA* fusion to monitor the T3SS expression in OE1-1 and screened AroG1 as one candidate with impact on the T3SS expression (Zhang et al., 2013). We therefore generated the *aroG1* and *aroG2* mutants to ascertain whether they affected T3SS expression in sucrose medium (*hrp*-inducing medium). Consistent with that of transposon mutants, the *popA* expression in *aroG1* mutant (RQ5687) was significantly reduced (112 versus 298 Miller Units of RK5050), while no alteration in *aroG2* mutant (RQ5803), and complementary *aroG1* fully restored the reduced *popA* expression to that of RK5050 (Figure 2A). The *popA* expression was further reduced in *aroG1/2* mutant (RQ5806) (76 versus 298 Miller Units), and complementary *aroG1* fully restored the reduced *popA* expression to that of RK5050 (Figure 2A). These results indicates that both AroG1 and AroG2 are cooperatively essential for T3SS expression in *R. solanacearum*. The AroG1 plays a major role on T3SS expression, while AroG2 becomes to function in the absence of AroG1.

AroG1 and AroG2 control biosynthesis of SA and AAA, and we investigated whether this reduced *popA* expression was due to the deficient of SA or AAA. Supplementary SA and AAA did not alter the *popA* expression in RQ5687 (Δ*aroG1*) and RQ5806 (Δ*aroG1/2*) (Figure 2B), indicating that impact of AroG1 and AroG2 on T3SS is not mediated with their downstream products of AAA and SA.

AroG1 and AroG2 are highly conserved in RSSC and we investigated whether they affected the T3SS expression in GMI1000, which exhibits different phenotypes from OE1-1 in tobacco plants. Consistent with that in OE1-1, the *popA* expression in GF0032 (GMI1000, Δ*aroG1*) and GF0034 (GMI1000, Δ*aroG1/2*) was significantly reduced, but not in GF0033 (GMI1000, Δ*aroG2*) (Supplementary Figure S2). As expected, the AroG1 of OE1-1 fully restored the reduced *popA* expression in GF0032 and GF0034 to that of wild-type strain (Supplementary Figure S2), confirming that the impact of AroG1 and AroG2 on T3SS is conserved in RSSC and the AroG1 of OE1-1 is functionally equivalent to that of GMI1000.

### Impact of AroG1 on T3SS Is Mediated Through the PrhA Signaling Cascade

**FIGURE 3:**
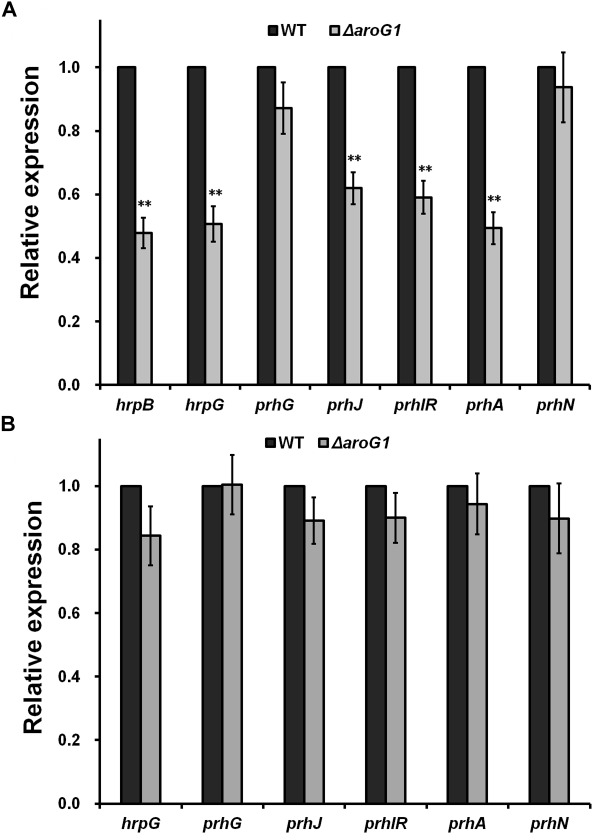
Relative expression of genes involved in T3SS regulation with *aroG1* deletion in **(A)** PS, **(B)** B medium. Dark bars, reporter strains (the control); gray bars, *aroG1* mutants corresponding to each reporter strain. Cells were grown to an OD600 of about 0.1 and subjected for *β*-galactosidase assay. Enzymatic activities with *aroG1* deletion was divided with that of control and relative values were presented. Mean values of four independent trials with three replications per trial were averaged and presented with SD (error bars). Statistical significance between the control and *aroG1* mutants was assessed using a *post hoc* Dunnett test following ANOVA. Significance level, ^∗∗^ indicates *P* < 0.01 (*t*-test).

The T3SS of *R. solanacearum* is directly controlled by the master regulator HrpB, and deletion of *aroG1* significantly impaired the *hrpB* expression in sucrose medium (Figure 3A), confirming that the impact of AroG1 on T3SS is mediated through the HrpB. Two close paralogs of HrpG and PrhG positively regulate the *hrpB* expression in a parallel way. Deletion of *aroG1* significantly impaired the *hrpG* expression only in sucrose medium, but not *prhG* expression in either rich or sucrose medium (Figure 3A,B). PrhN positively regulates the *prhG* expression, while its expression was not affected with the deletion of *aroG1* in either of media (Figure 3A,B). The *hrpG* expression is regulated by the well-characterized PrhA signaling cascade, including PrhA, PrhI/R and PrhJ. Interestingly, their expression was significantly impaired with the *aroG1* deletion in sucrose medium (Figure 3A,B), indicating that the impact of AroG1 on T3SS is mediated with the well-characterized PrhA-PrhI/R-PrhJ-HrpG signaling cascade.

### AroG1 and AroG2 Are Cooperatively Essential for Pathogenicity of *R. solanacearum* in Different Host Plants, While the AroG1 Plays a Major Role

**FIGURE 4:**
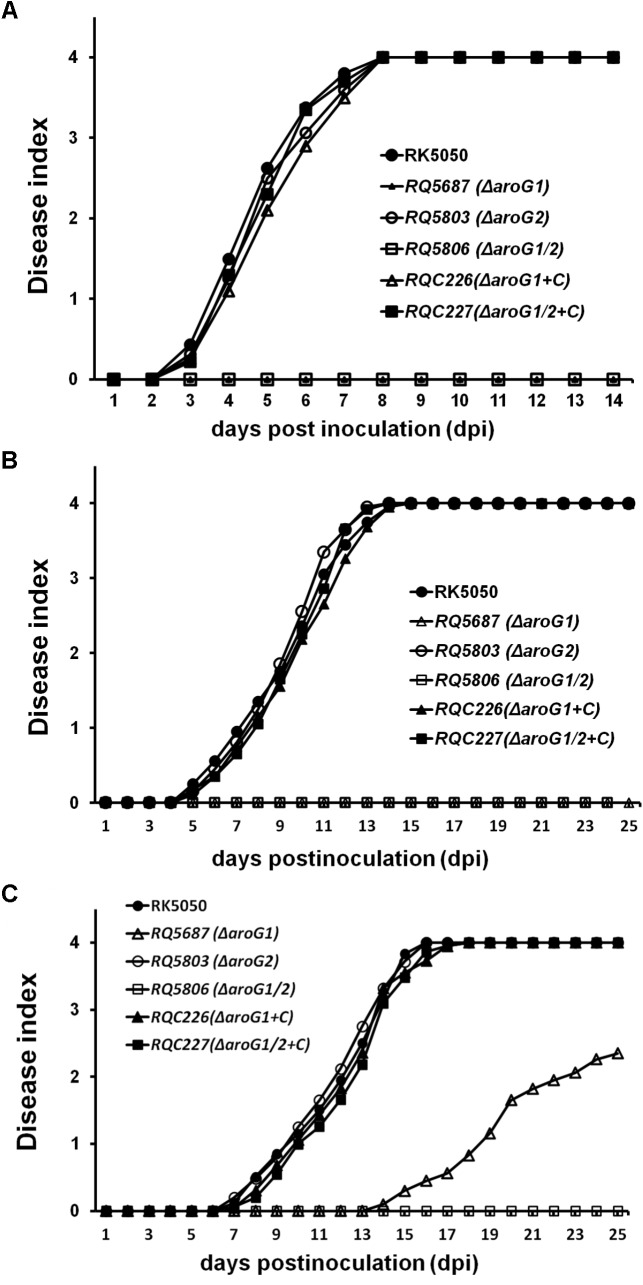
Pathogenicity test of *aroG* mutants on tomato plants **(A,B)** and tobacco plants **(C)**. **(A)** Petiole inoculation, about 3 μl of bacterial suspension at 10^8^ cfu ml^-1^ was dropped onto freshly cut surface of petioles; **(B)** soil-soaking inoculation, a bacterial suspension was poured into the soil of plants at a final concentration of 10^7^ cfu g^-1^ of soil; **(C)** leaf infiltration in tobacco leaves, about 50 μl of bacterial suspension at 10^8^ cfu ml^-1^ was infiltrated into tobacco leaves with a blunt-end syringe. Plants were inspected daily for wilt symptoms, and scored on a disease index scale from 0 to 4 (0, no wilting; 1, 1–25% wilting; 2, 26–50% wilting; 3, 51–75% wilting; and 4, 76–100% wilted or dead). RQC226 and RQC227 refer to *aroG1* and *aroG1/2* mutants complemented with *aroG1*, respectively. Each assay was repeated in at least four independent trials and each trial contains at least 12 plants. Mean values of all results were averaged with SD.

The T3SS is essential for pathogenicity of *R. solanacearum* in host plants and we investigated whether AroG1 and AroG2 play roles on pathogenicity. RK5050 invaded and killed tomato plants at about 13 days post inoculation (dpi) with the soil-soaking inoculation and 7 dpi with petiole-inoculation. RQ5803 (Δ*aroG2*) exhibited almost identical virulence as RK5050 in tomato plants with both inoculation methods, while RQ5687 (Δ*aroG1*) and RQ5806 (Δ*aroG1/2*) completely lost pathogenicity in tomato plants with both inoculation methods, and complementary *aroG1* fully restored their diminished virulence to that of RK5050 (Figure 4A,B).

When challenged tobacco plants with inoculation methods of soil-soaking (data not shown) and leaf infiltration (Figure 4C), RQ5803 (Δ*aroG2*) exhibited identical virulence as RK5050, while RQ5806 (Δ*aroG1/2*) completely lost the pathogenicity in tobacco plants (Figure 4C). RQ5687 (Δ*aroG1*) was avirulent on tobacco plants with soil-soaking (data not shown), while it exhibited significantly less virulence than RK5050 in leaf- infiltrated tobacco plants, which killed about half tobacco plants at 25 dpi (Figure 4C). Complementary *aroG1* fully restored the diminished or impaired virulence of *aroG1* or *aroG1/2* mutants in tomato and tobacco plants (Figure 4A–C), indicating that the AroG1 and AroG2 are cooperatively essential for pathogenicity of *R. solanacearum* in different host plants. The AroG1 plays essential role for pathogenicity in both tomato and tobacco plants, while the AroG2 is capable to partially function for pathogenicity in tobacco plants in the absence of AroG1.

### Expression of *aroG1* and *aroG2* Was Greatly Increased With Deletion of the Other

Despite high similarity, AroG1 and AroG2 exhibited different properties on bacterial growth and pathogenicity. We firstly investigated whether this difference was due to their different promoter activities. Fusions of *aroG1-lacZYA* and *aroG2-lacZYA* were introduced into different strains, and their promoter activities were evaluated with the *β*-galactosidase assay. Deletion of *aroG2* greatly increased the *aroG1* expression to about 15-fold higher level in rich medium and 10-fold higher level in limited medium compared with those in wild-type strains (Table 2). The *aroG2* expression was also greatly increased with *aroG1* deletion by about 20-fold higher levels in both media, while the expression of *aroG1* and *aroG2* was not altered with deletion of themselves (Table 2). The expression of *aroG1* and *aroG2* was also greatly increased in *aroG1/2* mutants, which was much less than those with deletion of single *aroG1* and *aroG2* (Table 2).

**Table 2:** Expression of *aroG1* and *aroG2* in different genetic backgrounds.

Strain	Genotype	*β*-galactosidase activity (Miller Units)			
		
		B medium		Sucrose medium	
			
		-Phe	++Phe	-Phe	+Phe
RQC212	OE1-1, *aroG1-lacZYA*	47 (9)	40 (8)	46 (11)	38 (9)
RQC214	OE1-1, *aroG2-lacZYA*	49 (7)	41 (9)	54 (13)	46 (8)
RQC216	Δ*aroG1, aroG1-lacZYA*	58 (12)	51 (7)	67 (14)	54 (11)
RQC218	Δ*aroG2, aroG1-lacZYA*	702 (29)	316 (43)	499 (25)	223 (28)
RQC228	Δ*aroG1, aroG2-lacZYA*	1072 (131)	353 (51)	989 (101)	316 (52)
RQC220	Δ*aroG2, aroG2-lacZYA*	84 (18)	55 (12)	73 (15)	49 (11)
RQC264	Δ*aroG1/2, aroG1-lacZYA*	469 (27)	219 (21)	409 (23)	199 (24)
RQC266	Δ*aroG1/2, aroG2-lacZYA*	706 (16)	311 (28)	743 (15)	347 (26)


*Cells were grown in medium to an OD600 of approximately 0.1 and subjected for *β*-Galactosidase assay. L-Phe was supplemented into both media at a concentration of 1 mM (+Phe) for enzyme assay. Mean values of four independent trials and three replications per trial were averaged and presented with SD in parentheses.*

DAHP synthases are usually sensitive to their corresponding AAA. For instance, the AroG is Phe-sensitive in *E. coli* (11). In the present study, L-Phe was supplemented into rich and limited media at a concentration of 1 mM to evaluate its feedback impact on *aroG* expression. Expression of *aroG1* and *aroG2* was significantly repressed with supplementary L-Phe in both media (Table 2), confirming that AroG1 and AroG2 are also Phe-sensitive in *R. solanacearum*. In consideration of the fact that AroG1 and AroG2 are cooperatively essential for AAA biosynthesis, growth and pathogenicity in *R. solanacearum*, this bacterium can greatly initiate the expression level of *aroG1* and *aroG2* in the absence of the other. And hence, the highly expressed AroG1 can alone fulfill the growth and pathogenicity, and highly expressed AroG2 is capable to partially substitute the function of AroG1.

### Neither Promoters nor the Unique N-Terminal Region in AroG2 Are Not Crucial Cause of Functional Difference Between AroG1 and AroG2

**FIGURE 5:**
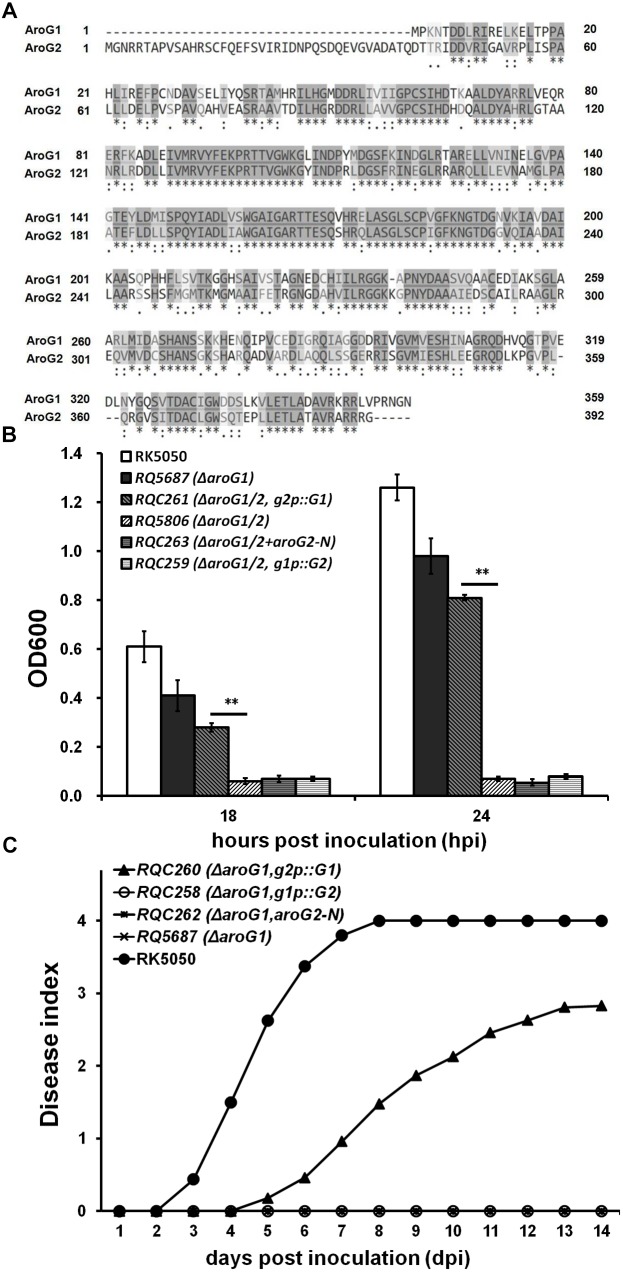
Functional characterization of mutated *aroG1*/*aroG2* in different backgrounds. **(A)** ClustalW analysis of AroG1 and AroG2 (58% identity and 88% similarity), **(B)** growth assay and **(C)** virulence assay of *aroG* mutants with mutated *aroG1*/*aroG2*. The N-terminal region of 43 AA is unique in AroG2 and this region was deleted to generate the truncated AroG2-N. The *g2p::G1* fused promoter of *aroG2* with CDS of *aroG1*, and *g1p::G2* fused promoter of *aroG1* with CDS of *aroG2*. The *g2p::G1, g1p::G2* and AroG2-N was integrated into chromosome of *aroG1* and *aroG1/2* mutants, respectively. Cells were inoculated into sucrose medium and OD600 was measured at 18 and 24 hpi for growth assay. Tomato plants were inoculated with petiole inoculation for virulence assay. Mean values of four independent trials and each trial contains at least 12 plants were averaged and presented with SD. Significance level, ^∗∗^ indicates *P* < 0.01 (*t*-test)

A remarkable structural difference between the AroG1 and AroG2 is a N-terminal region of 43 AA, which is unique in AroG2 (Figure 5A). We firstly investigated whether this structural difference is responsible for their functional difference. We deleted this unique N-terminal region of 43 AA in AroG2 and evaluated its contribution on growth and virulence in *aroG1* and *aroG1/2* mutants. Whereas this truncated AroG2-N failed to restore the diminished growth of *aroG1/2* mutant (RQ5806) in limited medium (Figure 5B), or the diminished virulence of *aroG1* mutant (RQ5687) in tomato plants (Figure 5C), confirming that this unique N-terminal region in AroG2 is not the cause for AroG2 to function weakly for growth and pathogenicity.

We thus transferred to investigate whether their promoters are cause for functional difference between the AroG1 and AroG2. Above promoter activity assay showed that the promoter activity of *aroG2* was greatly increased with the *aroG1* deletion, which was much higher than that of *aroG1* with the *aroG2*deletion. We therefore generated the promoter-exchanged *aroG1* and *aroG2* and evaluated their contribution on growth and virulence in *aroG1* and *aroG1/2* mutants. The fusion of *g2p::G1*, fused promoter of *aroG2* with CDS of *aroG1*, can partially restore the diminished growth of *aroG1/2* mutant in limited medium (Figure 5B), and the diminished virulence of *aroG1* mutant in tomato plants, which killed about half of petiole-inoculated tomato plants at 14 dpi (Figure 5C). Whereas the fusion of *g1p::G2*, fused promoter of *aroG1* with CDS of *aroG2*, failed to restore the diminished growth of *aroG1/2* mutant in limited medium, or the diminished virulence of *aroG1* mutant in tomato plants (Figure 5B,C), indicating that different promoter activities between *aroG1* and *aroG2* are not crucial cause for their functional difference on growth and pathogenicity.

### Both AroG1 and AroG2 Are Cooperatively Important for *in planta* Growth of *R. solanacearum*, While the AroG1 Plays a Major Role

**FIGURE 6:**
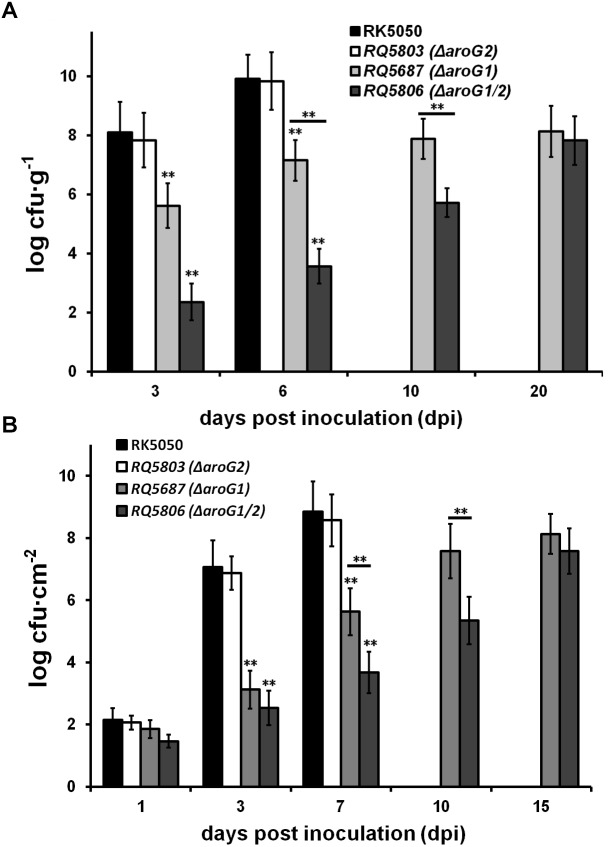
Growth assay of *aroG* mutants *in planta* of **(A)** tomato stems and **(B)** tobacco leaves. Stem species were removed from petiole-inoculated tomato plants, leaf disks were punched from leaf-infiltrated tobacco plants, and subjected for quantification of cells number by dilution plating, periodically. Mean values of four independent trials and each trial contains four plants were averaged and presented with SD (error bars). Statistical significance between RK5050 and *aroG* mutants or between *aroG1* and *aroG1/2* mutants was assessed using a *post hoc* Dunnett test following ANOVA. Significance level, ^∗∗^ indicates *P* < 0.01 (*t*-test).

Extensive proliferation in xylem vessels is one of the main virulence determinants of *R. solanacearum*, and we investigated whether AroG1 and AroG2 are required for the *in planta* growth of *R. solanacearum*. The wild-type strains cause petiole-inoculated wilted and died at 3 and 7 dpi, respectively, and we quantified their growth in tomato stems at 3 and 6 dpi, respectively, which reached to approximately 10^8^ cfu g^-1^ at 3 dpi, and reached to the maximum of approximately 10^10^ cfu g^-1^ at 6 dpi (Figure 6A). The *aroG2* mutant was same virulent as wild-type strains in tomato plants, and it exhibited identical growth as wild-type strains in tomato stems at 3 and 6 dpi (Figure 6A). The *aroG1* and *aroG1/2* mutants did not cause disease in tomato plants and we quantified their growth in tomato stems till to 20 dpi. The *aroG1* mutant proliferated to approximately 10^5^ cfu g^-1^ at 3 dpi, increased slowly to the maximum of approximately 10^9^ cfu g^-1^ at 10 dpi, and remained this density till to 20 dpi (Figure 6A). The *aroG1/2* mutant exhibited significantly less proliferation than *aroG1* mutant in tomato stems, which proliferated to approximately 10^2^ cfu g^-1^ at 3 dpi and increased slowly to the maximum of approximately 10^9^ cfu g^-1^ at 20 dpi (Figure 6A).

Growth of these mutants was also evaluated in tobacco leaves, which was infiltrated with cells suspension at a low concentration of 10^4^ cfu ml^-1^. Proliferation of the wild-type strains started from approximately 10^2^ cfu cm^-2^ at 1 dpi and increased daily to approximately 10^9^ cfu cm^-2^ at 7 dpi, when infiltrated leaves wilted and dried (Figure 6B). Both the *aroG1* and *aroG1/2* mutants proliferated slowly from approximately 10^2^ cfu cm^-2^ at 1 dpi to approximately 10^8^ cfu cm^-2^ at 15 dpi, when tobacco leaves became yellow (Figure 6B). Growth of the *aroG1/2* mutants in tobacco leaves was significantly less slowly than that of *aroG1* mutants (Figure 6B).

### Both AroG1 and AroG2 Are Cooperatively Important for the *in planta* Expression of T3SS in *R. solanacearum*, While the AroG1 Plays a Major Role

**FIGURE 7:**
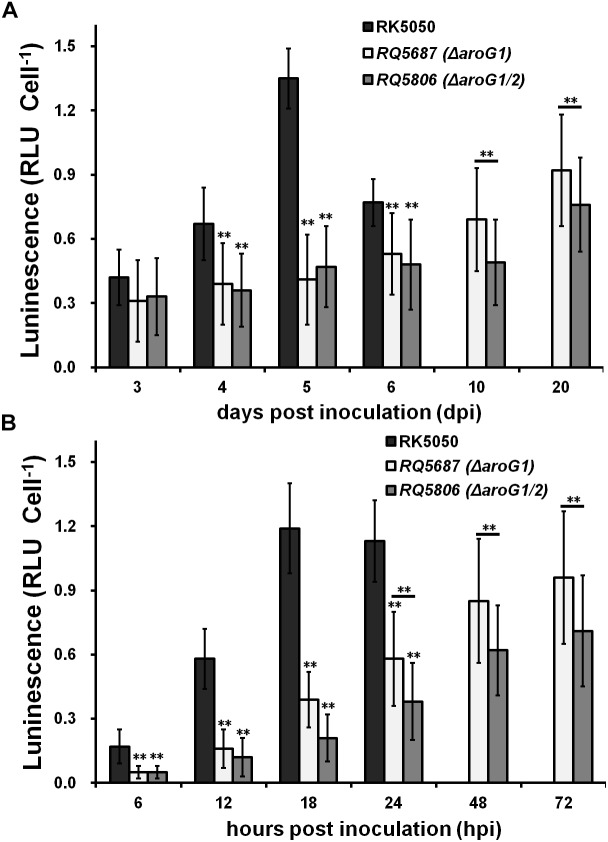
Expression of *popA* of *aroG* mutants *in planta* of **(A)** tomato stems and **(B)** tobacco leaves. Tomato plants were inoculated with petiole inoculation and stem species were removed periodically for enzyme assay with Galacto-Light Plus kit. Enzyme assay of RK5050 was performed till 6 dpi, when tomato plants became wilted and died, while that of *aroG1* and *aroG1/2* mutants was performed till 20 dpi since tomato plants remained healthy. Tobacco leaves were inoculated with leaf infiltration and leaf disks were punched periodically for enzyme assay. Enzyme assay of RK5050 was performed till 24 hpi, when tobacco leaves became wilted, while that of *aroG1* and *aroG1/2* mutants was performed till 72 hpi since tobacco leaves remained healthy. Cells number was quantified by dilution plating and luminescence was evaluated using GloMax20 luminometer (Promega). Enzymatic activity was presented with luminescence normalized with cells numbers. Mean values of four independent trials with four replications per trial were averaged and presented with SD. Significance level, ^∗∗^ indicates *P* < 0.01 (*t*-test).

Expression of the T3SS can be enhanced to about 20-fold higher level *in planta* than that *in vitro* (*hrp-*inducing medium), and we evaluated whether AroG1 and AroG2 are required for the T3SS expression *in planta*. The T3SS expression in wild-type strain (RK5050) was assayed till to 6 dpi in tomato stems, when plants withered and died, which reached to the maximum at 5 dpi and decreased rapidly at 6 dpi (Figure 7A). The T3SS expression in *aroG1* and *aroG1/2* mutants increased slowly till to 20 dpi, which was significantly less than that of RK5050 in tomato stems (Figure 7A). Different from that in *hrp-*inducing medium, the *aroG1* and *aroG1/2* mutants exhibited almost identical T3SS expression in tomato stems (Figure 7A).

The T3SS expression in RK5050 was assayed up to 24 hpi in tobacco leaves, when tobacco leaves became wilting, which increased slowly and reached to the maximum at about 18 hpi (Figure 7B). The T3SS expression in *aroG1* and *aroG1/2* mutants was assayed up to 72 hpi, which increased slowly and reached to the maximum at 72 hpi, but they were significantly less than that of RK5050 in tobacco leaves (Figure 7B). Different from that in tomato stems, the *aroG1/2* mutant exhibited significantly less T3SS expression than *aroG1* mutant in tobacco leaves (Figure 7B).

### AroG1 and AroG2 Are Not Required for the HR Elicitation of GMI1000 in Tobacco Leaves

Deletion of *aroG1* and *aroG2* significantly impaired the *in planta* expression of T3SS, which is responsible for the HR elicitation of GMI1000 in tobacco leaves, and we investigated whether GMI1000 requires AroG1 and AroG2 for HR elicitation. Cells suspension at 10^8^ cfu ml^-1^ was infiltrated into tobacco leaves with a blunt-end syringe and development of necrotic lesions was investigated periodically. It was intriguing that both *aroG1* and *aroG1/2* mutants exhibited identical development of necrotic lesions as GMI1000 in tobacco leaves (Supplementary Figure S3), indicating that AroG1 and AroG2 are not required for the HR elicitation of GMI1000 in tobacco leaves.

## Discussion

In the present study, we provided genetic evidence to demonstrate that two putative DAHP synthases of AroG1 and AroG2 are cooperatively essential for biosynthesis of AAA in *R. solanacearum*. Three isoenzymes of DAHP synthases, AroF, AroG and AroH, have been identified in many bacteria to control the first step in the shikimate pathway (Herrmann, 1983; Herrmann and Weaver, 1999; Sprenger, 2006). Only the AroG was annotated in genomes of RSSC strains. The *aroG1*/*2* mutants were indeed auxotrophic in limited medium, and supplementary AAA or SA significantly restored the diminished growth of *aroG1*/*2* mutants in limited medium, confirming that AroG1 and AroG2 are involved in the shikimate pathway and thus are responsible for AAA biosynthesis in *R. solanacearum*. The shikimate pathway can also lead to production of some AAA-derivatives, such as vitamin K, folic acid and ubiquinone, which are also important for bacterial growth (Dosselaere and Vanderleyden, 2001; Gosset et al., 2001). It can explain the fact that growth recovery with SA was much better than that with AAA. Supplementary SA and AAA can just partially restore diminished growth of *aroG1/2* mutants in limited medium, indicating that AroG1 and AroG2 might be involved in the production of some novel compounds, which are also important for growth.

The shikimate pathway is a common route in microorganisms and plants that leads to production of AAA and derivatives (Herrmann and Weaver, 1999; Sprenger, 2006). AAA and some derivatives can be detected in apoplastic and xylem extracts of tomato plants, and *R. solanacearum* can metabolize these compounds inside tomato plants and facilitate it to thrive in tomato plants (Zuluaga et al., 2013). It is consistent with the fact that the *aroG1/2* mutants failed to grow in limited medium, but grew slowly in host plants. Extensive proliferation in xylem vessels is one of the most important virulence determinants of *R. solanacearum* in host plants (Roberts et al., 1988; Denny, 1995). It was as expected that the *aroG1* and *aroG1/2* mutants exhibited completely diminished or significantly weakened virulence in host plants since their proliferation was significantly impaired in tomato and tobacco plants. Growth of *aroG2* mutants was not altered in tomato and tobacco plants, and they exhibited identical virulence as the wild-type strain in host plants. Tobacco plants exhibit different metabolic activities on secondary metabolites, i.e., salicylic acid, from tomato plants (Bellés et al., 1999, 2006). Moreover, different host plants usually display different symptoms, depending upon infecting strains (Lin et al., 2008). It can explain the fact that the *aroG1* mutants display different phenotypes on different host plants that completely lost the virulence in tomato plants, but remained weakened virulence in tobacco plants.

The T3SS is another essential virulence determinants of *R. solanacearum* (Vasse et al., 2000; Genin et al., 2005), which was significantly impaired in *aroG1* and *aroG1/2* mutants both *in vitro* and *in planta*. It is consistent with above virulence results that *aroG1* and *aroG1/2* mutants exhibit completely diminished or significantly weakened virulence in host plants. The T3SS expression was not altered in *aroG2* mutants either *in vitro* or *in planta*. It is as expected that *aroG2* mutants exhibited identical virulence as wild-type strains. The AroG2 seems not to function for bacterial growth, T3SS expression and virulence in the presence of AroG1. In consideration of facts that the *aroG1/2* mutants display enhanced phenotypes on bacterial growth, T3SS expression and virulence compared to the *aroG1* mutants, the AroG2 is capable of carrying out part of functions of AroG1 in the absence of AroG1. The *aroG1* and *aroG1/2* mutants grew slowly *in planta* that eventually got to the maximal densities of approximately 10^8^ cfu g^-1^ in tomato stems and 10^8^ cfu cm^-2^ in tobacco leaves, respectively. Note that tomato plants become wilting when *R. solanacearum* proliferates to a density of about 10^8-9^ cfu g^-1^ in stems and tobacco leaves become withered at about 10^7-8^ cfu cm^-2^ (Zhang et al., 2011, 2013). Whereas the *aroG1* and *aroG1/2* mutants failed to cause any wilting symptom in tomato plants even they reached to this high density at 10–20 dpi, indicating that these slow growth might enable host plants to initiate effective resistance reaction leisurely, or AroG1 and AroG2 are also required for some novel virulence determinants.

The *hrpB* expression was significantly impaired in *aroG1* mutant, which is consistent with the fact that HrpB directly controls the entire T3SS (Cunnac et al., 2004; Tamura et al., 2005). The *hrpB* expression is positively regulated by two close paralogs of HrpG and PrhG in a parallel way (Zhang et al., 2013), while only the *hrpG* expression was significantly impaired with the *aroG1* deletion in sucrose medium, suggesting that the impact of AroG1 on T3SS is mediated through the HrpG-HrpB pathway, but independent of PrhG. Expression of *prhA, prhIR*, and *prhJ* was significantly impaired in *aroG1* mutants, which form the well-known PrhA signaling cascade and positively regulate the *hrpG* expression in tandem, confirming that the impact of AroG1 on T3SS is mediated through the well-characterized PrhA-prhI/R-PrhJ-HrpG signaling cascade. Since the T3SS assembly and secretion of T3Es require a lo of energy, T3SS expression is not activated until *R. solanacearum* gets contact with some signals, i.e., host signals or mimic signals in nutrient-limited medium. The *aroG1/2* mutants failed to grow in limited medium, which should suffer some extreme conditions, at least the extreme starvation. *R. solanacearum* should stop all unessential activities, at least coordinating for energy conservation, while the T3SS expression was not completely diminished in the *aroG1/2* mutants under this extreme condition, which was remained to about a quarter of the wild-type strain. The T3SS should play some roles on the survival of *R. solanacearum* under these extreme conditions.

DAHP synthases are usually sensitive to their corresponding AAA (McCandliss et al., 1978; Umbarger, 1978; Herrmann, 1983; Pittard, 1996). Supplementary L-Phe can significantly repress the expression of *aroG1* and *aroG2* in *R. solanacearum*. This feedback repression results in relatively low expression levels of *aroG1* and *aroG2* in wild-type strains (about 50 Miller Units in both rich and limited media). The *aroG2* expression was greatly increased to about 20-fold higher level with *aroG1* deletion. We above concluded that both AroG1 and AroG2 cooperatively function for bacterial growth, T3SS expression and virulence. The AroG2 is capable of carrying out part of functions of AroG1 in the absence of AroG1, which might be due to greatly expressed AroG2 in the absence of AroG1. The *aroG1* expression was greatly enhanced to about 14-fold higher level with the *aroG2* deletion, and hence, the highly expressed AroG1 can alone fulfill growth, T3SS expression and pathogenicity in the absence of AroG2. The promoter activity of *aroG2* in *aroG1* mutants was much higher than that of *aroG1* in *aroG2* mutants, while the promoter of *aroG2* could just partially substitute native promoter for *aroG1* to fulfill the growth and pathogenicity. On the contrary, promoter of *aroG1* failed to enable AroG2 to fulfill the growth and pathogenicity, suggesting that functional difference between AroG2 and AroG1 should be due to their structural difference, but not the promoter activities. A remarkable structural difference between AroG2 and AroG1 is the unique N-terminal region of 43 AA in AroG2. Whereas the truncated AroG2 failed to restore the growth and pathogenicity in *aroG1* mutants. Some AA residues have been characterized to be important for the function of DAHP isoenzymes (Jossek et al., 2001; Sprenger, 2006), indicating that certain AA residues are responsible for functional difference between AroG2 and AroG1.

Taken together, our genetic results demonstrated that two putative DAHP synthases of AroG1 and AroG2 are involved in the shikimate pathway and are responsible for the AAA biosynthesis in *R. solanacearum*. They cooperatively exhibit essential functions on bacterial growth, T3SS expression and pathogenicity. The AroG1 plays a major role on these phenotypes, while AroG2 is capable to partially carry out the function of AroG1 in the absence of AroG1.

## Author Contributions

YZ and KO conceived and designed the experiments. WZ and JL performed the experiments. XS, YH, KO, and YZ analyzed and discussed the results. YZ wrote and revised the manuscript.

## Conflict of Interest Statement

The authors declare that the research was conducted in the absence of any commercial or financial relationships that could be construed as a potential conflict of interest.

## Footnotes

1 https://iant.toulouse.inra.fr/bacteria/annotation/cgi/ralso.cgi
