# Supplementary material for: Functional Characterization of Two Putative DAHP Synthases of AroG1 and AroG2 and Their Links With Type III Secretion System in Ralstonia solanacearum
Source: Front Microbiol. 2019 Feb 12;10:183. doi: 10.3389/fmicb.2019.00183 (PMC6379268; doi:10.3389/fmicb.2019.00183)
Supplement: Supplementary file 1 [file Table_1.docx]

**Table S1. Primers used in this study**

| primer | sequence | Reference | |
| --- | --- | --- | --- |
| aroG1A1B | ATCGCTCAAGGACGTCACCA | | This study |
| aroG1B1C | GCCAGTGCGCGTGCGCGGGCGGAAAGCTCCCGGGGGATGT | | This study |
| aroG1A2C | ACATCCCCCGGGAGCTTTCCGCCCGCGCACGCGCACTGGC | | This study |
| aroG1B2H | CTAAGCTTCGGTTCGCCAGGGTTGTC | | This study |
| aroG2A1B | ATGGATCCTGAACTACGTCGCGAACATGACGG | | This study |
| aroG2B1C | CCCCTCGACGCGAGTCCCCGTGACGGCGGAAATCGGCGGG | | This study |
| aroG2A2C | CCCGCCGATTTCCGCCGTCACGGGGACTCGCGTCGAGGGG | | This study |
| aroG2B2H | TCAAGCTTAACGACACCAGCATGGCGCAAAGC | | This study |
| aroG1B3H | TCAAGCTTTCAGTTGCCGTTGCGGGGCA | | This study |
| aroG2B3H | CGAAGCTTGCTAGGCGCCAAACCAGCA | | This study |
| aroG1MuB1C | AGCTCTTTCAGTTCGCGGGTACCCAGGTCGTCGGTGTTCT | | This study |
| aroG1MuA2C | AGAACACCGACGACCTGGGTACCCGCGAACTGAAAGAGCT | | This study |
| aroG2MuB1C | CGCGGTACCCGGGGCAGTCCGCCGATT | | This study |
| aroG2MuA2C | CCGGGTACCGCGCACCGCTCGTGTTT | | This study |
| PG1::G2B1C | GCAGTCCGCCGATTGCCCATGGAAAGCTCCCGGGGGATGT | | This study |
| PG1::G2A2C | ACATCCCCCGGGAGCTTTCCATGGGCAATCGGCGGACTGC | | This study |
| PG2::G1B1C | TCGTCGGTGTTCTTCGGCATTGACGGCGGAAATCGGCGGA | | This study |
| PG2::G1A2C | TCCGCCGATTTCCGCCGTCAATGCCGAAGAACACCGACGA | | This study |
| PG1::G2dNB1C | ATGCGCACGTCATCGATGCGCATGGAAAGCTCCCGGGGGA | | This study |
| PG1::G2dNA2C | TCCCCCGGGAGCTTTCCATGCGCATCGATGACGTGCGCAT | | This study |
| PG2::G2dNB1C | ATGCGCACGTCATCGATGCGCATTGACGGCGGAAATCGGC | | This study |
| PG2::G2dNA2C | GCCGATTTCCGCCGTCAATGCGCATCGATGACGTGCGCAT | | This study |
| glmsdown | GCGCTCAAGCTCAAGGAGATC | Zhang *et al*., 2011 | |
| Tn7R | CACAGCATAACTGGACTGATTTC | Choi *et al*., 2005 | |
| lacZR1 | GCGCCATTCGCCATTCAGGCT | Zhang *et al*., 2013 | |
