# Supplementary figures and images for: Functional Characterization of Two Putative DAHP Synthases of AroG1 and AroG2 and Their Links With Type III Secretion System in Ralstonia solanacearum
Source: Front Microbiol. 2019 Feb 12;10:183. doi: 10.3389/fmicb.2019.00183 (PMC6379268; doi:10.3389/fmicb.2019.00183)

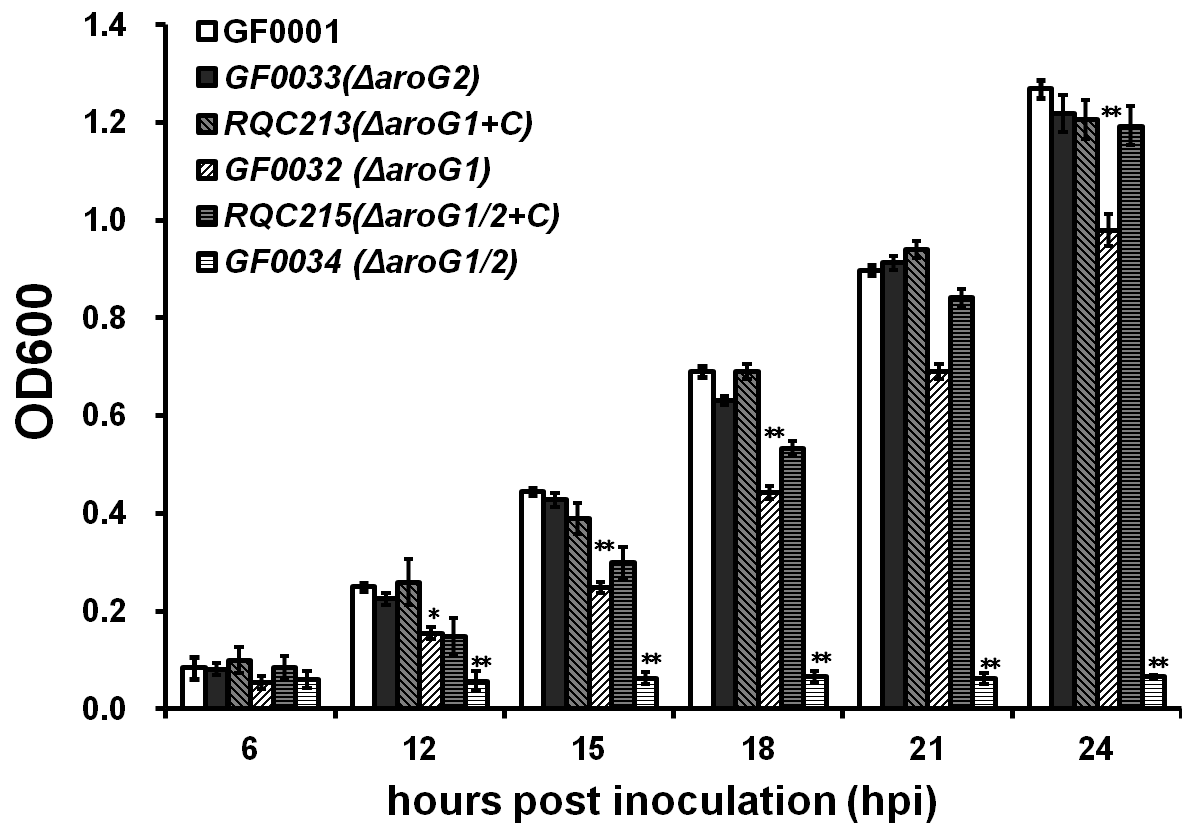

Supplement: Supplementary file 2 [file Image_1.tif]

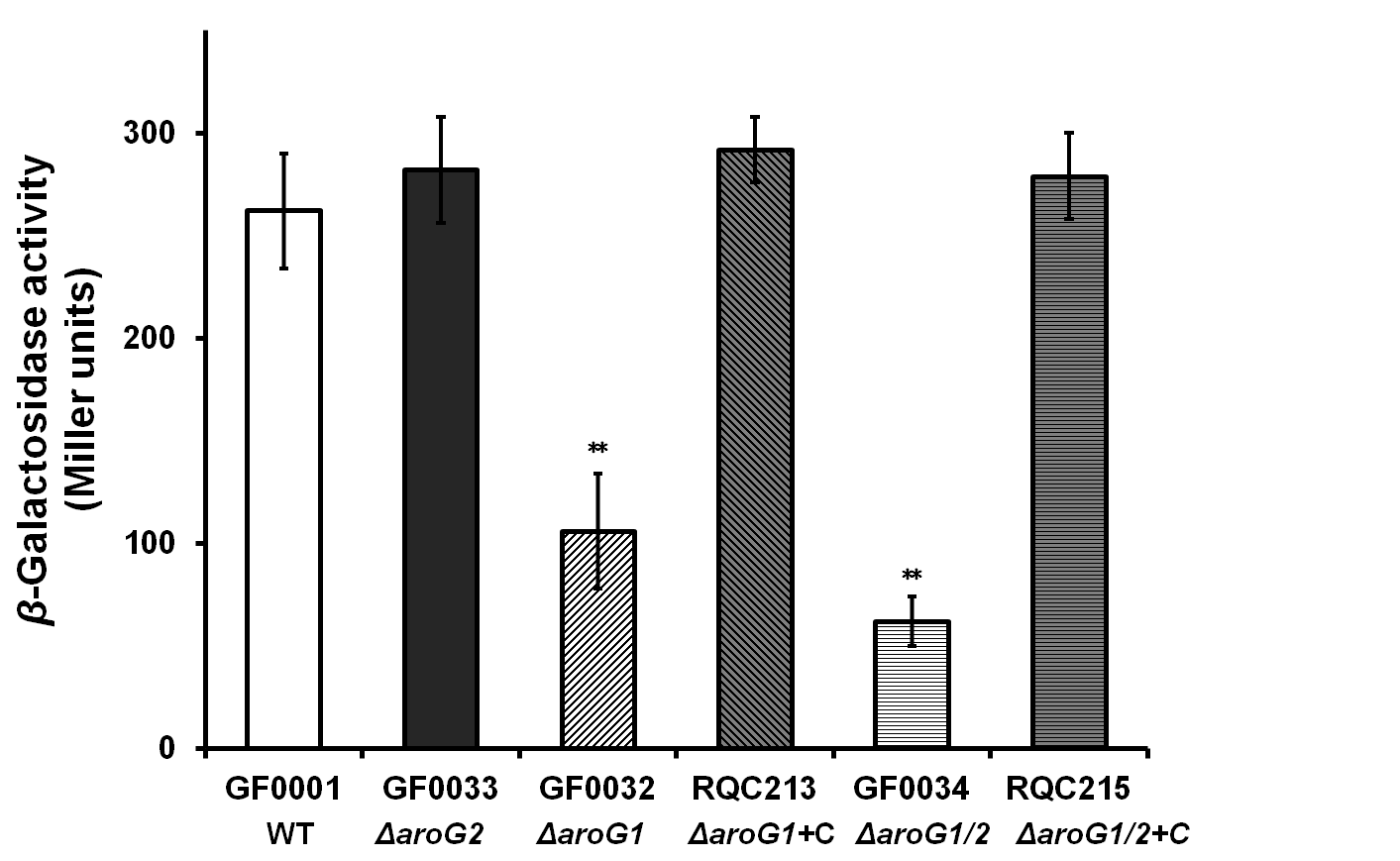

Supplement: Supplementary file 3 [file Image_2.tif]

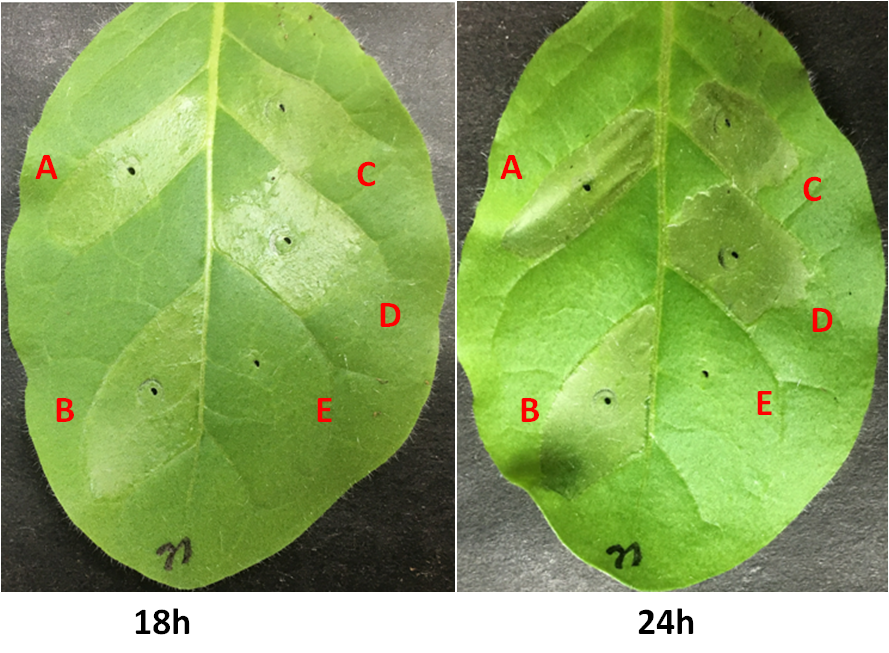

Supplement: Supplementary file 4 [file Image_3.tif]
